# The Role of Glucosamine and Chondroitin Sulfate in the Prevention of Colorectal Cancer: A Systematic Review

**DOI:** 10.7759/cureus.25401

**Published:** 2022-05-27

**Authors:** Asma A Khan, Vij Mannan, Muhammad Ahad Pervaiz, Aqsa Akram, Elina S Momin, Muhammad Sanusi, Tejasvi Kashyap, Abeer O Elshaikh

**Affiliations:** 1 College of Medicine, California Institute of Behavioral Neurosciences & Psychology, Fairfield, USA; 2 Internal Medicine, California Institute of Behavioral Neurosciences & Psychology, Fairfield, USA; 3 Urology, California Institute of Behavioral Neurosciences & Psychology, Fairfield, USA; 4 Internal Medicine, Dallah Hospital, Riyadh, SAU; 5 Internal Medicine, Smt. Nathiba Hargovandas Lakhmichand Municipal Medical College, Ahmedabad, IND; 6 Internal Medicine, Cardiology, Shenyang Medical College, Shenyang, CHN; 7 Internal Medicine, Cardiology, California Institute of Behavioral Neurosciences & Psychology, Fairfield, USA; 8 General Practice, California Institute of Behavioral Neurosciences & Psychology, Fairfield, USA

**Keywords:** dietary supplements, preventive practices, disability & cancer prevention, colorectal cancer, glucosamine, chondroitin sulfate

## Abstract

Currently, colorectal cancer is the third most common cancer in the world. Recently, glucosamine and chondroitin have gained popularity for their beneficial effects on cancer. They have already been recognized for their therapeutic role in osteoarthritis. This systematic review aims to analyze the relationship between the combined consumption of glucosamine and chondroitin and the prevention of colorectal cancer. Three databases: PubMed, Google Scholar, and Science Direct, were searched to collect relevant articles. After screening full-text articles, seven studies were included in the systematic review. The review found a supportive association between glucosamine and chondroitin and the decreased incidence of colorectal cancer. Through an anti-inflammatory effect on the cell signaling pathway, the supplementation caused a reduction in colorectal cancer occurrence. The dose, frequency of usage of the supplement, and weight of individuals, along with the use of non-steroidal anti-inflammatory drugs, also affected the efficacy. To further assess this relationship, it is necessary to conduct double-blind, randomized controls trials for the supplements in cancer prevention and further explore their safety and efficacy with different ethnicities, drugs, doses, and weight individuals.

## Introduction and background

Colorectal cancer is currently the third most common cancer globally, with an alarming number of 1.9 million new cases detected in 2020 [1]. About 10% to 11% of cancers diagnosed are colorectal cancers [1]. In 2018, the highest country ranked in its incidence was Hungary consisting of 51.2% of cancer patients for both males and females combined [1]. The risk of developing colorectal cancer in men is one in 23 and one in 25 for women [2].

**Figure 1:**
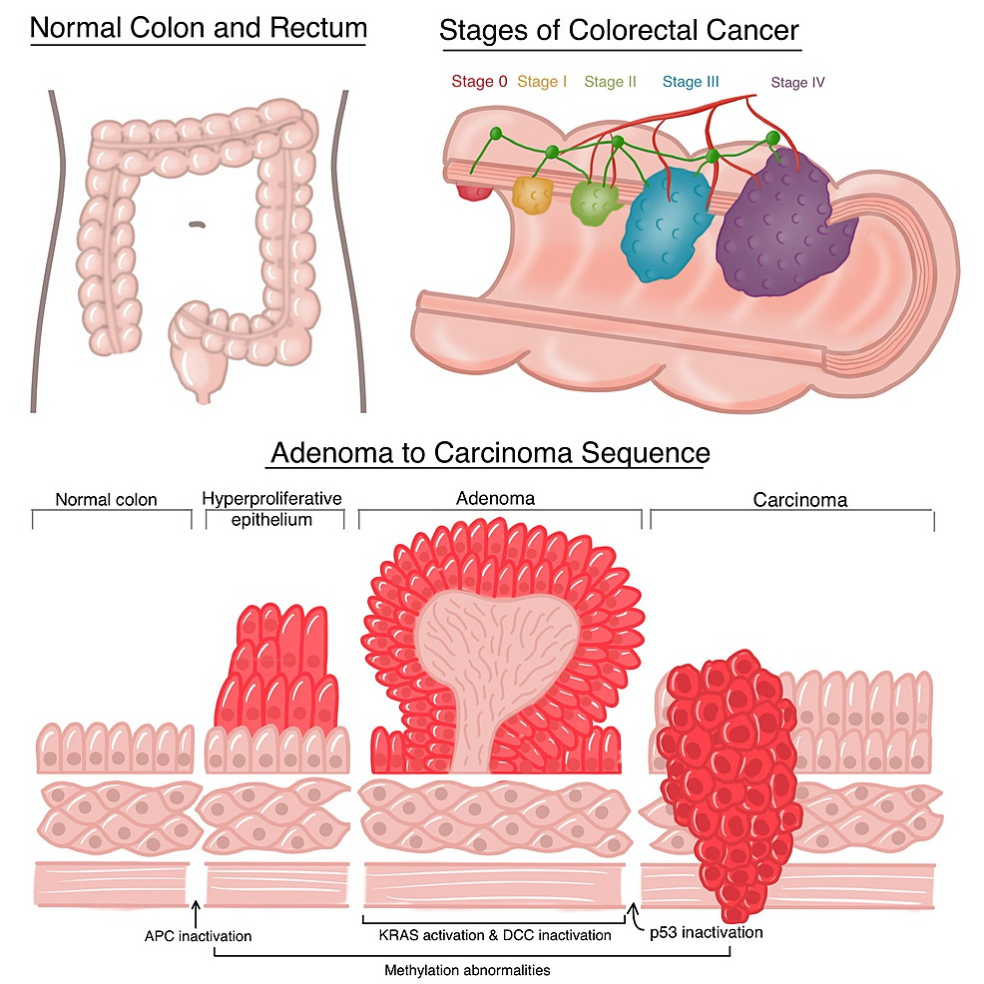
Comparison of the histological feature of normal colon cells, adenoma, and colorectal cancer cells. APC: Adenomatous polyposis coli, KRAS: Kirsten rat sarcoma virus, DCC: Netrin receptor DCC, P53:  tumor protein p53 Figure 1 is an original illustration by Tejasvi Kashyap.

The cancer is present in both the colon and rectum. Though these are two separate cancers, they are often grouped because of their similarity [3]. Through the help of screening methods, colorectal cancer can be detected in the early stages [4]. According to the U.S. Preventive Services Task Force, screening of medium to high-risk individuals above the age of 45 is considered beneficial in detecting and reducing mortality of colorectal cancer [5]. It is highly recommended for individuals between the age group of 50 to 75 years [5]. A stratified analysis predicts that the risk of women and African Americans being diagnosed with late-stage colorectal cancer is significantly higher than men and Caucasians [6]. In general, those living under low socioeconomic conditions have more chances of developing colorectal cancer than individuals belonging to privileged backgrounds [6]. The gold standard for screening is coloscopy, where adenoma detection can independently predict the occurrence of colorectal cancer [7]. Also, DNA fecal testing of individuals presenting with adenomas shows a high percentage of methylated genes CDKN2A, MGMT, and MLH1 compared to individuals with no polyps that can be used to determine the risk of colorectal cancer, as shown in Figure 1. [7]. Unfortunately, metastases are present in the primary diagnosis of about 20%-25% in colon cancer patients and 18% in rectal cancer patients [8]. It is detected by the growth on the lining of the colon or rectum called polyps which can change into cancer. Adenomatous polyps are a classification of polyps considered precursors for colorectal cancer because of their high risk of dysplasia [2]. Cancers usually begin from benign neoplasms, progressing into adenocarcinomas through histological changes for polyps/serrated adenomas [9]. Colorectal cancer can be classified from 0 to IV as determined by the Union of International Cancer Control based on the size of the tumor, the extent of metastases, and the spread through lymph nodes [10]. Another classification can be done through molecular and clinical catheterization through CMS 1 to CMS 4 [11]. The treatment varies according to the stages of cancer. Stage 0 and I can be treated by a colonoscopy procedure removing the local polyp [12]. For Stage II and III, removal of part of the colon and the surrounding lymph nodes through partial colectomy is performed, and Stage III patients undergo chemotherapy [12]. Stage IV involves ablation of removal of the colon and parts of the involved organs, along with chemotherapy [12]. Adjuvant treatment such as radiation or medication may be coupled alongside the treatment in cases with high metastases [10].

Dietary supplements have been reported and studied preciously to monitor their efficacy against the prevention of colorectal cancer. Glucosamine and chondroitin are dietary supplements derived from animal products and have been used for many years in patients suffering from osteoarthritis [13]. Glucosamine is categorized as a hexosamine sugar made by humans as a building block for connective tissue elements such as glycolipids, glycoproteins, and hyaluronic acid [14]. Chondroitin Sulfate is a type of glycosaminoglycan in cartilage known for its water-absorbing properties to counteract compressive forces exerted on the cartilage [14]. Increasing popularity has been seen over the years in the use of glucosamine and chondroitin as a supplement for various reasons [15]. Although an extensive systematic quality assessment has concluded the effectiveness of their role in the prevention of osteoarthritis, their efficacy in preventing colorectal cancer has not yet been determined [16]. Because the supplements are not required to undergo official Food and Drug Administration (FDA) screening, the safety of their consumption is unclear as side effects have been reported by various consumers [17].

Some studies promote glucosamine and chondroitin as a preventative tool for serrated polyps and colorectal adenoma, considered precursor lesions to colorectal cancer [18]. The objective of the following systematic review is to explore the effect of glucosamine and chondroitin consumption on the incidence of colorectal cancer.

## Review

Methodology

Study Protocol

The Preferred Reporting Items for Systematic Review and Meta-Analyses (PRISMA) 2020 Guidelines [19] were referred to execute and record the data presented in this systematic review. 

Sources of Data Collection

Three databases were used to collect relevant articles: PubMed, ScienceDirect, and Google Scholar. Each database was intensively screened using the keywords: glucosamine, chondroitin, and colorectal cancer. 

Search Strategy 

The use of Medical Subject Headings (MeSH) was applied to stratify the search strategy on PubMed further. For other databases, keywords were used to collect relevant articles. 

((Glucosamine) OR (D-glucosamine) OR ("Glucosamine/administration and dosage"[Mesh]) OR ("Glucosamine/therapeutic use"[Mesh)] AND ((chondroitin) OR (chondroitin) OR ("Chondroitin/administration and dosage"[Mesh]) AND ((colorectal cancer)*

Table 1 summarizes the keywords and search results used in each database, respectively. 

**Table 1:** Keywords and search strategy

Database	Keywords	Search Results
PubMed	Search Strategy applied*	123
Google Scholar	Glucosamine, Chondroitin, Colorectal cancer	230
ScienceDirect	Glucosamine, Chondroitin, Colorectal cancer	337

Inclusion and Exclusion Criteria

The review conducted included all types of articles from all locations of the world. There was no limit on the age of literature or population size. All grey literature and animal studies were excluded, and only articles in English were acquired. Solely those studies monitoring the use of both glucosamine and chondroitin simultaneously were collected. Selected patients were those who reported the consumption of glucosamine and chondroitin together. The intervention was glucosamine and chondroitin, and the control group had individuals who did not take glucosamine and chondroitin supplementation lastly, the outcome was colorectal cancer. 

Data Extraction

The relevant studies were screened and collected by two independent researchers, A.K and V.M, anonymously through the Rayyan Software [20]. The intervention and outcome were closely monitored. The data extracted from the studies were classified according to the author, year of publication, study type, study design, results, conclusion, participants, intervention time, and intervention dosage.

Risk and Quality Assessment

Each study included in the systematic review evaluated the risk and quality assessment. The Newcastle Ottawa Scale was used for all observational studies. The Revised Cochrane’s Risk of Bias Tool was administered for randomized controlled trials. The studies meeting the criteria of > 70% for quality and grade were selected for the systematic review. 

Results

**Figure 2:**
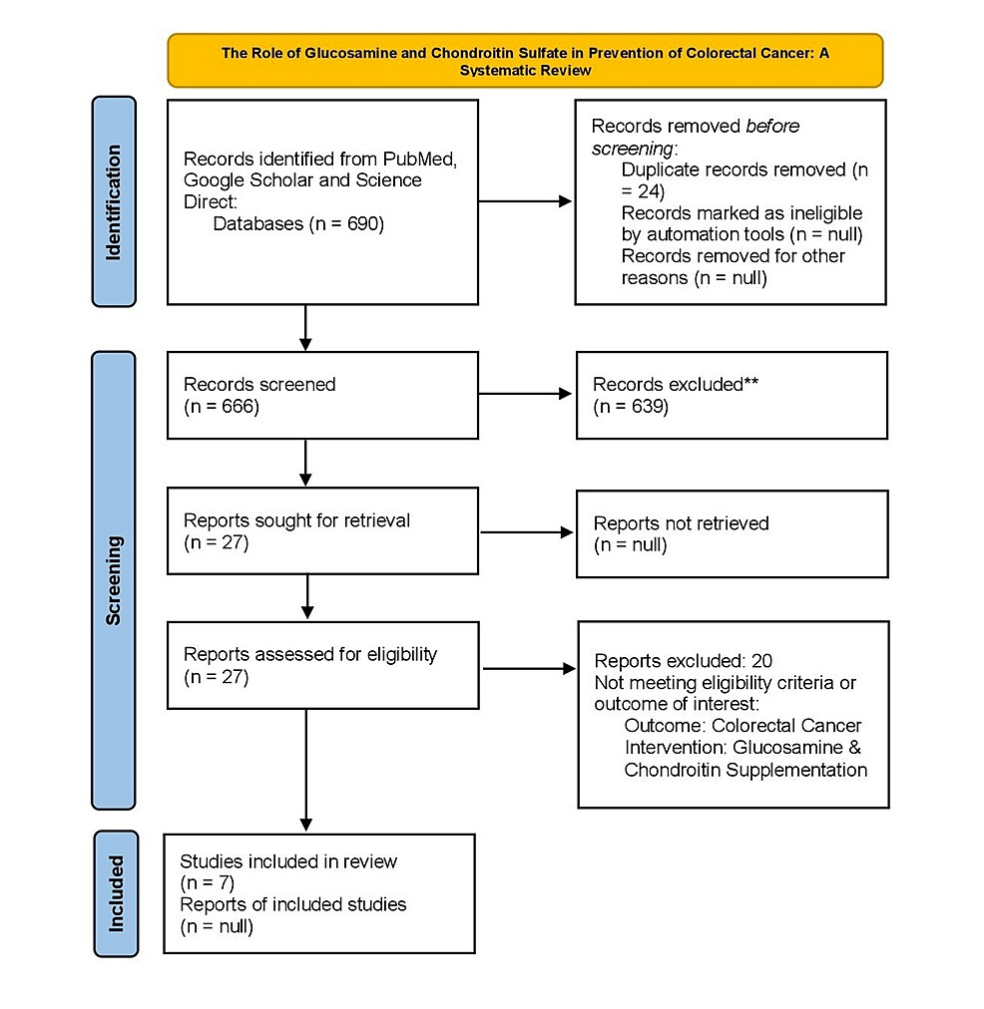
The PRISMA 2020 flow diagram PRISMA: Preferred Reporting Items for Systematic Reviews and Meta-Analyses

After applying the search strategy and keywords from the three databases, 690 studies were collected. PubMed had 123 studies, 337 from ScienceDirect, and 230 from Google Scholar. The studies were entered into Rayyan [20] and scanned for duplicates. After duplicate removal of 24 studies, 666 studies were carefully screened by the abstract and title available. Of those, 639 did not match the previously determined inclusion and exclusion criteria. Twenty-seven studies were selected and assessed for eligibility through the full text. The included studies were a total of seven, as the remaining 20 were excluded because of no outcome of interest. A complete PRISMA flow diagram was created and is presented below as Figure 2 [19]. 

Discussion

Recent studies have assessed the relationship between glucosamine and chondroitin in their role of preventing colorectal cancer. The influence of the supplements on the cell signaling pathways and genes has been analyzed. The age, ethnicity, and body mass index (BMI) stratification were observed in its use. Also, the duration of exposure and the amount of glucosamine and chondroitin consumed daily was correlated with the prevention of colorectal cancer. 

The Anti-Inflammatory Properties of Glucosamine and Chondroitin

**Figure 3:**
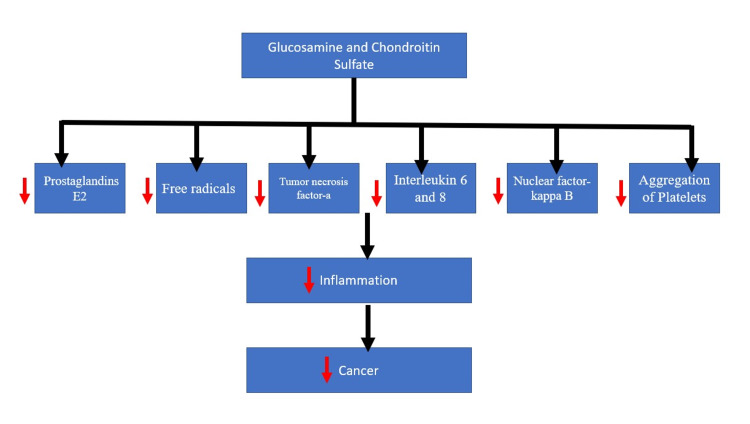
Anti-inflammatory mechanism of glucosamine and chondroitin sulfate.

Glucosamine and chondroitin are considered to have anti-inflammatory properties through various mechanisms leading to anticancer effects mentioned in Figure 3 [21]. Glucosamine is known to prevent the interleukin cell cascade and expression of specific genes inhibiting both catabolic and anabolic pathways, resulting in decreased cancer-promoting activities [21]. The damage caused to endothelial cells and surrounding tissue can elicit cancer formations [21]. With both glucosamine and chondroitin consumption, the reduction of colorectal cancer and lung cancer can be seen through decreased inflammatory processes [21]. The factors included are the production of prostaglandins E2, free radicals of oxygen and nitrogen, and cytokines such as tumor necrosis factor-a and interleukin-6 [21]. Glucosamine sulfate also lowers the nuclear factor Kappa B when observed in vitro in cartilage forming chondrocytes [21].

In the Ibanez-Sanz et al. 2020 case-control trial, 25,811 colorectal cancer patients were screened and compared with a control group yielding statistically significant results of (OR:0.80; 95% CI, 0.72-0.88) [22]. The use of non-steroidal anti-inflammatory drugs with the supplementation of glucosamine and chondroitin has proven to cause an additive relationship against the prevention of colorectal cancer [22]. The underlying mechanism implies the involvement of the COX-2 gene promotor being closely located on the same attachment site of nuclear factor kappa B [22]. 

The Bell et al. study monitored a significant decrease in the mortality rate of participants who reported glucosamine and chondroitin with the prevalent decrease in the occurrence of cancer because of an additional mechanism of inhibition of the aggregation platelets [23].

Similarly, the follow-up study by Kantor et al. in 2016 monitored 121,700 registrations through questionnaires and favored the relationship with colorectal cancer by observing the risk ratio (RR: 0.77; 95% CI: 0.58-0.999) [24]. It concluded the results based on the prevention of the signaling cascade of nuclear factor Kappa B because of the inhibition of the breakdown process of subunit ik-B [24]. Glucosamine is considered to decrease systemic inflammation through the reduced use of mRNA. The involvement of reducing the interleukin-8 was also involved [24].

Efficacy and Safety

The effectiveness was assessed for the use of glucosamine and chondroitin in the individuals taking them. The safety was evaluated of both supplements before participant collection.

The use of sulfated glucosamine is considered more effective because the sulfate provides enhanced synovial fluid strength and increased production of glycosaminoglycans [21]. The consumption of sulfated glucosamine is more prevalent among individuals, and the underlying mechanism could be the possible reason for its reduced effects on the occurrence of colorectal and lung cancer [21].

Kantor et al.'s 2013 cohort study with a baseline of 2000 to 2002 and a follow-up in 2008 observed a reduced risk of colorectal cancer in individuals but not significant enough to be considered to have an independent relationship with the decreased risk in the prevention of colorectal cancer [25]. Confounding factors such as education, regular screening through colonoscopy, use of other multivitamins, and a history of other medical diseases could have a possible role in the potency of glucosamine and chondroitin consumption [25].

A beneficial association has been observed between the use of both glucosamine and chondroitin together with non-steroidal anti-inflammatory drugs [22]. When the supplements are paired with non-steroidal anti-inflammatory drugs, the results show a statistical significance in the prevention of colorectal cancer [22]. Furthermore, enforcement of health-promoting behaviors such as regular exercise, being physically active, not smoking, and limiting alcohol use could decrease the chance of colorectal combined with the help of glucosamine and chondroitin [26].

In the Ibáñez-Sanz et al., 2018 study, a case-control, and meta-analysis of the role of glucosamine and chondroitin both with univariate analysis and multivariate when observing their efficacy with the use of non-steroidal anti-inflammatory drugs [27]. An 18% reduction was observed in total mortality compared with individuals who did not use glucosamine and chondroitin. A 13% decrease was observed in cancer risk [27]. With the combined use of aspirin, the following supplementation can significantly affect the prevention of colorectal cancer [27]. However, numerous adverse effects have been reported with the use of supplements. Glucosamine is considered to cause changes in the skeletal muscle, causing insulin resistance leading to problems for diabetics and increasing hypersensitivity for people with selfish allergies [27]. Mild gastrointestinal disturbances were also reported with chondroitin use, such as diarrhea and nausea [27]. Glucosamine may also increase bleeding if combined with additional drugs [21].

Despite glucosamine and chondroitin not having significant safety screenings, they have been shown to provide synergistic benefits when combined with anti-inflammatory produces such as non-steroidal anti-inflammatory drugs. Combining the practice of healthy lifestyle behaviors with supplementation also indicates a higher chance of prevention of colorectal cancer.

Dose and Duration of Use

The dose and frequency of the intake of glucosamine and chondroitin can significantly impact the chances of preventing colorectal cancer. Variation in the participants’ supplement consumption was seen within the studies themselves.

The Satia et al. study was a cohort study assessing individuals with the use of glucosamine and chondroitin within 10 years to determine their impact on lung cancer and colorectal cancer [21]. Ibanez-Sanz et al., in 2020, based their study on the recommended daily dose of 1200 mg for chondroitin sulfate and 1500 mg for glucosamine [22]. The average duration of consumption was considered to be 90 days for exposure [22]. The non-steroidal anti-inflammatory drugs used were inspected for 100 days for consumption daily with glucosamine and chondroitin supplementation [22]. The use of the supplementation for more than 12 months or 90 days on the daily dosage showed decreased risk, and those who reported using the supplement with non-steroidal anti-inflammatory drugs for more than 240 days found significant benefits against colorectal cancer [22]. A dose and duration-dependent relationship were established. For the Conway et al. study, the dosage was inferred according to the European Union of Law [26]. 

The Bell et al. was a cohort study monitoring patients from 2000 to 2002 and then a follow-up in 2008 [23]. About 97% of the participants took the supplement for a minimum of four days per week [23]. They also reported that 42% of the participants had taken it for a minimum of three years or more [23].

Similarly, in the Kantor et al. 2013 study, although the results did not reach statistical significance, a reduction of 45% with a p-value of 0.16 was seen for colorectal cancer when both glucosamine and chondroitin were consumed together for more than four days per week with a duration of more than three years, especially when compared to non-users [25]. Though the dose was also non-significant among users, more association was established for individuals consuming a high dosage [25].

The following studies show a positive correlation between the frequency of use when both glucosamine and chondroitin are used together. However, the relationship with dosage has not been clearly observed due to it not being mentioned clearly in some of the included studies, but the consumption of 1200 mg of chondroitin sulfate and 1500 mg of glucosamine daily with non-steroidal anti-inflammatory drugs does show to have anti-cancer effects. 

The Age and Weight Groups Benefitting From Glucosamine and Chondroitin

The effects of glucosamine and chondroitin were both monitored. They yielded a protective impact for those individuals at an older age and within a higher range of BMI compared to those who were underweight and middle-aged.

Bell et al., 2012, and Satia et al., 2009, studies focused on participants in the age range of 50 to 76 [23]. Both studies reported a reduction of risk and indicated a beneficial relationship between glucosamine and chondroitin consumption in the particular age group [23].

The Ibáñez-Sanz et al. 2018 study had a mean population of 64.6 years and 44.4% women [27]. Even then, the study only showed a positive preventive effect despite having fewer women and younger participants as compared to other studies when coupled with the use of non-steroidal anti-inflammatory drugs [27].

A cross-sectional study conducted by Conway et al. reported increased use of glucosamine and chondroitin as a preventative measure against colorectal cancer and used as a joint supplement for osteoarthritis [26]. The majority of the population were female and of Caucasian ethnicity. The study indicated more extensive supplement use among women [26]. Glucosamine and chondroitin observed a more significant decrease in C-reactive protein, especially in females [21]. The implication indicates a better response by the female gender to the use of glucosamine and chondroitin because of the decreased pro-inflammatory cytokines present [21].

The Ibanez-Sanz et al. study in 2020 screened 25,811 colorectal cases and compared them with 129,117 controls from the duration of 2010 to 2015 [22]. The correlation between the benefit of glucosamine and chondroitin with the group with a BMI of greater than or equal to 25 kg/m^2 had more of a reduced risk of developing colorectal cancer than the group categorized under the category of having a BMI of less than 25 kg/m^2 [22].

In the Kantor et al. 2013 study, the inverse relationship was determined among participants in the obese or overweight category and the incidence of colorectal cancer with the use of both glucosamine and chondroitin with a p-value of 0.02 [25]. However, there was no association between underweight and normal-weight individuals and the prevention of colorectal cancer [25]. The obese category benefited more from glucosamine and chondroitin supplementation effects because of the high amount of inflammation previously present in their body that decreased after the supplement was ingested [25]. In contrast, the Kantor et al.'s 2016 follow-up study on healthcare professionals reported a higher association between the use of glucosamine and chondroitin in preventing colorectal cancer in individuals with BMI less than 25 kg/m^2 category, with a risk ratio of 0.55, as compared to individuals in the obese and overweight category, with a risk ratio of 0.91 [24]. Though the difference is non-significant, with a p-value of 0.09, there is a marked variation in results seen among the two categories [24]. The results could differ because of the small group of obese individuals present in the study [24]. Also, mechanisms not dependent on inflammation, such as hyperinsulinemia, are more likely to be present in obese rather than lean individuals [24].

The above-mentioned studies indicate an increased response to the consumption of glucosamine and chondroitin against the occurrence of colorectal if individuals are above 50, overweight, and female. 

The following Table 2 includes details about all studies relevant to the use of glucosamine and chondroitin in the role of colorectal cancer.

**Table 2:** Data Extraction Table of all studies included in the systematic review. NSAID: non-steroidal anti-inflammatory drugs, ATC: anatomical therapeutic chemical

Author	Study Type	Study Design	Results	Conclusion	Participant	Intervention time	Intervention dosage
Bell et al. – 2012 [23]	Cohort	77,510 participants between the ages of 50 to 76 were enrolled in the cohort through a questionnaire from 2000 to 2002 on glucosamine and chondroitin use. A mortality follow-up was done in 2008.	Glucosamine showed a significant impact on the prevention of cancer (HR: 0.87 95% CI: 0.76–0.98). Chondroitin was in 2/3 of the glucosamine supplements.	Glucosamine use was considered significant in decreasing death from breast, prostate, and colorectal cancer. Both were deemed to be prevalent in the decrease of mortality and diseases.	61,613 are participants in the control group. 1,093 were former users of chondroitin and glucosamine, and 4178 were current users of the supplement.	Used within the last 10 years	The majority took the supplement for at least four days per week for a minimum of three years
Kantor et al. – 2013 [25]	Cohort	A follow-up study monitored 75,137 participants enrolled between 2000 to 2002 in the year 2008.	Individuals using glucosamine with chondroitin for more than four days per week for three years or more had a 45% less risk of colorectal cancer than those who didn’t use the supplement. (HR: 0.55; 95 % CI 0.30–1.01; p-trend: 0.16).	A positive correlation between the use of glucosamine and chondroitin in the prevention of colorectal cancer is evident. The use of glucosamine alone did not yield significant results in the association of prevention in colorectal cancer.	59,024 are participants in the control group. 6,509 were low users of chondroitin and glucosamine, and 3,481 were frequent users.	They have been used for the last 10 years.	6,509 participants used the supplement for less than four days per week or less than three years. In comparison, 3,481 participants used it for equal or greater than four days per week or more than three years.
Kantor et al. – 2016 [24]	Cohort	A follow-up study with 121,700 registered healthcare workers data collection in 2002 and 2010 for the use of glucosamine and chondroitin supplementation and the occurrence of colorectal cancer through questionaries.	Only glucosamine and chondroitin used together showed a favorable outcome of risk (RR: 0.77; 95% CI: 0.58–0.999) for preventing colorectal cancer.	When the use of glucosamine is combined with chondroitin, it serves as a protective effect against colorectal cancer.	Glucosamine and chondroitin sulfate cases were 12,455, and the control group had 83,945 participants.	The frequency used between 2002 and 2010.	N/A
Satia et al. – 2009 [21]	Cohort	50 to 76-year-old individuals completed a questionnaire about supplements they consumed.	Glucosamine and chondroitin sulfate used in the last 10 years indicated a lower risk of colorectal cancer of HR: 0.73 (95% CI, 0.54-0.98) and HR: 0.65 (95% CI, 0.45-0.93) respectively.	Even with varying demographics, the reduction of both colorectal and lung cancer was seen.	Patients with colorectal cancer were 428, and the control group had 76,084 participants.	Used within the previous 10 years.	N/A
Conway, Ph.D. et al. – 2021 [26]	Cross-sectional survey	The National Health Services database was screened for diagnosed breast, prostate, or colorectal cancer patient survivors and contacted through electronic or phone services for data collection.	Glucosamine and chondroitin, classified as joint supplements, were consumed by 61 individuals (5.8%), of which 13 patients (6.1%) had colorectal cancer.	Dietary supplementation is commonly used and viewed as a preventative measure against cancer.	Case of colorectal cancer 214 of which 13 used glucosamine and chondroitin. 61 participants used glucosamine and chondroitin sulfate in the study.	Two years of follow up between 2015 and 2017.	Dosage according to the European Union of Law.
Ibanez-Sanz et al. – 2020 [22]	Case-Control Study	The SIDIAP database was used to screen colorectal cancer cases of 25,811 and 129,117 control from 2010 to 2015.	The use of chondroitin sulfate and glucosamine resulted in (OR: 0.83; 95% CI, 0.70–0.98), whereas both used along with non-steroidal anti-inflammatory drugs showed (OR: 0.80; 95% CI, 0.72–0.88).	Chondroitin and glucosamine consumed do not establish an independent relationship with colorectal cancer but, if used with non-steroidal anti-inflammatory drugs, yield a favorable outcome in preventing colorectal cancer.	Patients with colorectal cancer were 25,811, and the control group had 129,117 participants.	The supplements are used between less than 12 months and greater than 36 months.	Dosage of 1200 mg for chondroitin sulfate and 1500mg for glucosamine per day.
Ibáñez-Sanz et al. – 2018 [27]	Case-Control Study and Meta-analysis	Interviews of 2140 cases of colorectal cancer and 3950 population controls were conducted on demographic and drug uses.	The univariate analysis of chondroitin sulfate and glucosamine (CG) use showed a 53% reduction in colorectal cancer (OR: 0.47; 95% CI: 0.28–0.79), but the multivariate analysis showed no signs of (adjusted OR: 0.82; 95% CI: 0.47–1.40). The difference could be attributed to the adjustments made to NSAID use.	Though there is no prevalent relationship between the independent use of chondroitin and glucosamine in preventing colorectal cancer, the additional use of NSAID showed a favorable outcome.	Patients with colorectal cancer were 2140, and the control group had 3950 participants.	N/A	Participants using glucosamine with the ATC Code: M01AX05 and chondroitin sulfate with the ATC code: M01AX25 were only included. Glucosamine daily dose is 1.5g.

Limitations

The systematic review has certain limitations. Firstly, a relatively small number of studies were included in the review. Secondly, only articles available in the English language were screened. Other articles in different languages were excluded irrespective of their eligibility. Also, only observational studies were found where participants either self-reported the data through questionnaires or were contacted through electronic means for interviews. There was a lack of clinical trials in the review. Lastly, the overweight individuals' sample size was relatively small. 

All these limitations should be kept into consideration when evaluating the results of this systematic review. 

## Conclusions

The systematic review aimed to explore the relationship established between the combined use of glucosamine and chondroitin with the incidence of colorectal cancer. Most of the studies included demonstrated a positive relationship between the consumption of supplements and the prevention of colorectal cancer. Variations were seen in individuals consuming glucosamine and chondroitin in either higher frequencies, increased weight, or simultaneous use of non-steroidal anti-inflammatory drugs. The review enforces the need for double-blind, randomized control trials including a larger group of individuals in the overweight category, with diverse ethnical groups and anti-inflammatory drugs to monitor the effects of glucosamine and chondroitin sulfate and determine their role in the prevention of colorectal cancer. 
